# Risk factors for the exacerbation of patients with 2019 Novel Coronavirus: A meta-analysis

**DOI:** 10.7150/ijms.47052

**Published:** 2020-07-06

**Authors:** Jinqiu Zhao, Xiaosong Li, Yao Gao, Wenxiang Huang

**Affiliations:** 1 Department of Infectious Diseases, The First Affiliated Hospital of Chongqing Medical University, Chongqing, China, 400016.; 2 Laboratory medicine, The First Affiliated Hospital of Chongqing Medical University, Chongqing, China, 400016.

**Keywords:** 2019 novel coronavirus, pneumonia, risk factor, exacerbation prediction, meta-analysis

## Abstract

Many studies have reported the risk factors for exacerbations in patients with 2019 novel coronavirus (2019-nCoV). This study aims to perform the meta-analysis of risk factors for the exacerbation of the novel coronavirus-infected pneumonia (NCIP). PubMed, Embase and Google scholar have been searched. We included the cohort studies involving risk factors for the exacerbation of NCIP. This meta-analysis compared the risk factors of patients between intensive care (ICU) group and non-ICU group. Two cohort studies were included in this study. After comparing the patients between intensive care (ICU) group and non-ICU group, several important factors were found to significantly increase the risk of exacerbations in patients with NCIP, and they included hypertension (RR=2.34; 95% CI=1.21 to 4.51; P=0.01), cardiovascular diseases (RR=2.28; 95% CI=1.13 to 4.58; P=0.02), COPD (RR=7.65; 95% CI=1.24 to 47.13; P=0.03), dyspnea (RR=2.89; 95% CI=2.05 to 4.08; P<0.00001), myalgia or fatigue (RR=1.24; 95% CI=1.01 to 1.52; P=0.04), but several factors such as gender, Huanan Seafood Wholesale Market exposure, diabetes, chronic liver disease, malignancy, fever, cough, expectoration, headache and diarrhoea appeared to have no obvious effect on the exacerbation of pneumonia. In addition, as the exacerbation of pneumonia, some complications had the high probability to occur according to the meta-analysis of acute respiratory distress syndrome (ARDS) (RR=13.95; 95% CI=6.20 to 31.41; P<0.00001), shock (RR=24.29; 95% CI=4.66 to 126.69; P=0.0002), acute cardiac injury (RR=10.32; 95% CI=3.05 to 34.96; P=0.0002) and acute kidney injury (RR=5.90; 95% CI=1.32 to 26.35; P=0.02) between two groups. Several risk factors were confirmed to significantly improve the risk of exacerbation in patients with NCIP, which was very important for the exacerbation prediction and treatment of these patients.

## Introduction

In December 2019, the novel coronavirus-infected pneumonia (NCIP) was first found in Wuhan, Hubei Province, China 1-4. With the ongoing spring festival travel in China and the worldwide travel, the numbers of NCIP patients have been rapidly increased 5. On January 3, 2020, the 2019 novel coronavirus (2019-nCoV) was identified to result in NCIP, and was a distinct clade from the beta-coronaviruses associated with human severe acute respiratory syndrome (SARS) and Middle East respiratory syndrome (MERS) 6.

According to the first 425 confirmed NCIP in Wuhan from December 2019 to January 2020, the epidemiologic characteristics confirmed human-to-human transmission among close contacts since the middle of December 2019 and revealed that the epidemic was doubled in size every 7.4 days in the early stage 3. The human-to-human transmission of NCIP was also confirmed by case reports 7, 8. The novel coronavirus was found in stool samples of patients with abdominal symptoms, indicating that fecal-oral transmission might occur for NCIP 9.

One cohort study conducted in Jin Yin-tan Hospital (Wuhan, China) first reported the epidemiological, clinical, laboratory and radiological characteristics, as well as clinical outcomes in 41 NCIP patients 10. The clinical features mainly included fever, cough, dyspnea, myalgia, fatigue, sputum production, headache, haemoptysis, and diarrhoea. Some underling comorbidities may have some effect on the exacerbation of NCIP, and they included diabetes, hypertension, cardiovascular disease, chronic obstructive pulmonary disease (COPD), malignancy and chronic liver disease etc 11-14. In addition, the exacerbations of some cases commonly led to organ dysfunction such as shock, acute respiratory distress syndrome (ARDS), acute cardiac injury, acute kidney injury and even death 10, 15. Another cohort study also systematically reported the epidemiological and clinical features of 138 patients with NCIP in Zhongnan Hospital of Wuhan University (Wuhan, China) 12.

It is widely accepted that severe cases receive intensive care unit (ICU) care with non-severe cases do not receive ICU care. This study aimed to compare the epidemiological and clinical features between ICU patients and non-ICU patients in order to find the risk factors for exacerbation prediction of NCIP patients, which was very valuable to identify the development, treatment and prognosis of NCIP patients. However, the aforementioned two cohort studies reported some conflicting results such as hypertension, cardiovascular disease and diabetes 10, 12. Therefore, this meta-analysis was conducted to investigate the risk factors for exacerbation prediction of NCIP patients.

## Methods

Ethical approval and patient consent were not required because this was a meta-analysis of previously published studies. Two investigators independently searched the following databases (inception to February 8 2020): PubMed, Embase and Google scholar. The electronic search strategy was performed using with the following keywords: “novel coronavirus” and “clinical features” or “clinical characteristics”. The following inclusive selection criteria were applied: (i) patients were diagnosed with NCIP; (ii) study design was the cohort study comparing ICU patients with non-ICU patients.

We used a piloted data-extraction sheet, and collected the following information: publication year, first author, number of patients, age and gender in two groups. Data were extracted independently by two investigators. This meta-analysis focused on the risk factors including baseline characteristics, signs and symptoms, complications on the exacerbations of NCIP. Furthermore, risk factors should be compared between ICU patients and non-ICU patients in all included cohort studies.

Risk ratio (RR) with 95% confidence intervals (CI) was used for all dichotomous outcomes. The random-effects model was used regardless of heterogeneity which was assessed by I^2^ statistic. I^2^ > 50% indicated significant heterogeneity 16. Sensitivity analysis was needed when encountering significant heterogeneity. P<0.05 suggested statistically significance between two groups. All analyses were conducted using Review Manager Version 5.3.

## Results

Two cohort studies involving 179 patients were included in this study 10, 12. Two descriptive studies were excluded 17, 18. One study published in Chinese that did not compare the epidemiological and clinical features between severe cases and non-severe cases was also removed 19. Among the two included cohort studies, one was the retrospective, single-center case series of 138 patients in Zhongnan Hospital of Wuhan University (Wuhan, China) from January 1 to January 28, 2020 12, and another study involved 41 patients in Jin Yin-tan Hospital (Wuhan, China) by January 2, 2020 10.

The main characteristics of the two cohort studies were presented in Table 1. One study demonstrated that patients in ICU group was significantly older than those in non-ICU group (66 (57-78) versus 51 (37-62), median (IQR), P<0.001) 12, but the age range between two groups was not statistically different in another study (49.0 (41.0-61.0) versus 49.0 (41.0-57.5), median (IQR), P=0.60) 10. In addition, these two studies showed similar gender and Huanan Seafood Wholesale Market exposure. Among the total 179 patients, 49 cases (27.37%) received the ICU care while the remaining 130 cases (72.63%) did not need the ICU care.

### Baseline characteristics for exacerbation

**Figure 1:**
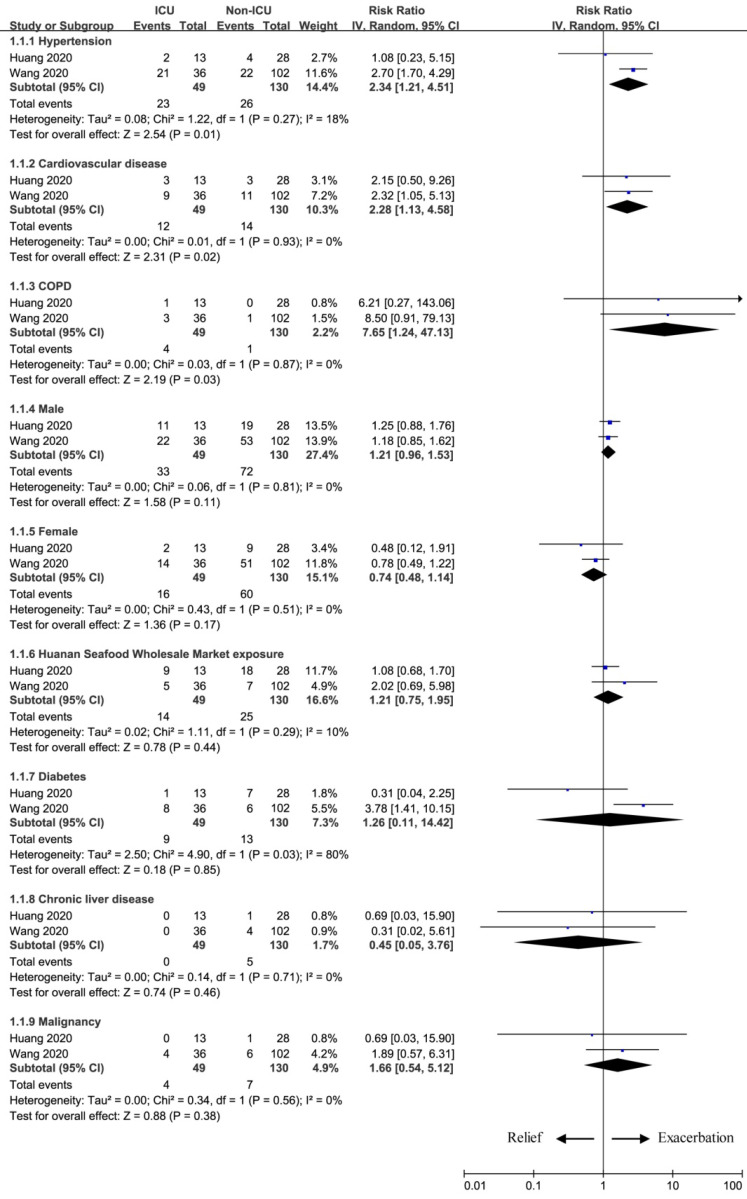
The influence of baseline characteristics on exacerbation.

We conducted the meta-analysis of the most common characteristics for exacerbation of 2019 Novel Coronavirus-Infected Pneumonia (Figure 1). These factors included hypertension, cardiovascular diseases, chronic obstructive pulmonary disease (COPD), gender (male/female), Huanan Seafood Wholesale Market exposure, diabetes, chronic liver disease and malignancy.

Patients with hypertension had the higher risk to obtain ICU care than those without hypertension (RR=2.34; 95% CI=1.21 to 4.51; P=0.01). Another two factors also improved the risk for ICU care between two groups in terms of cardiovascular diseases (RR=2.28; 95% CI=1.13 to 4.58; P=0.02) and COPD (RR=7.65; 95% CI=1.24 to 47.13; P=0.03). However, other factors showed no obvious effect on the exacerbation of novel coronavirus between two groups, and they included male (RR=1.21; 95% CI=0.96 to 1.53; P=0.11), female (RR=0.74; 95% CI=0.48 to 1.14; P=0.17), Huanan Seafood Wholesale Market exposure (RR=1.21; 95% CI=0.75 to 1.95; P=0.44), diabetes (RR=1.26; 95% CI=0.11 to 14.42; P=0.85), chronic liver disease (RR=0.45; 95% CI=0.05 to 3.76; P=0.46) and malignancy (RR=1.66; 95% CI=0.54 to 5.12; P=0.38) between two groups.

### Signs and symptoms for exacerbation

**Figure 2:**
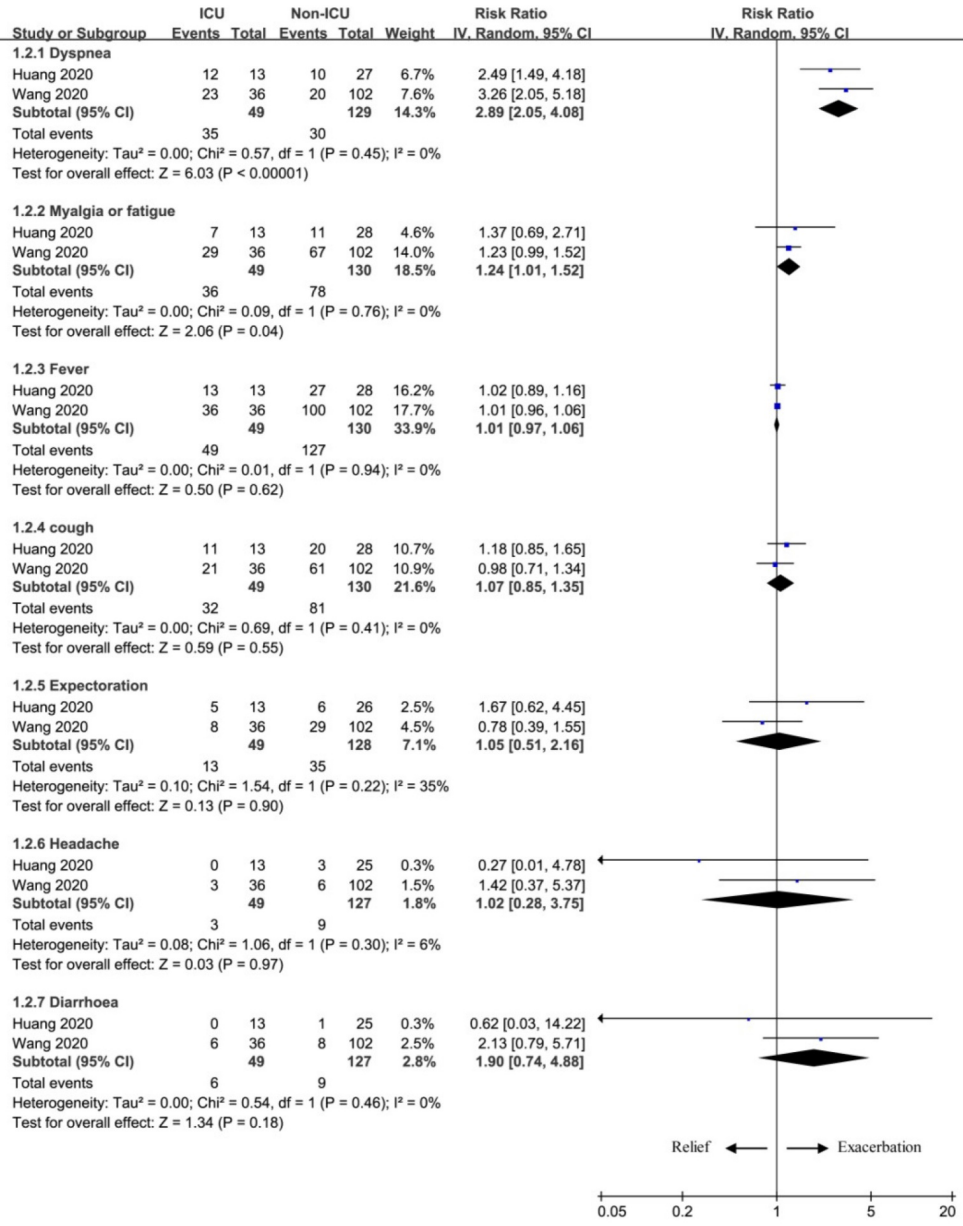
The influence of signs and symptoms on exacerbation.

The common signs and symptoms of NCIP mainly included fever, cough, myalgia or fatigue, dyspnea, expectoration, headache and diarrhea. This meta-analysis helped to identify some signs and symptoms for the exacerbation prediction of pneumonia (Figure 2).

Two important symptoms were found to substantially increase the number of patients receiving ICU care, and included dyspnea (RR=2.89; 95% CI=2.05 to 4.08; P<0.00001), myalgia or fatigue (RR=1.24; 95% CI=1.01 to 1.52; P=0.04). In contrast, several signs and symptoms had no remarkable effect on the exacerbation of NCIP, because there was no statistical difference of fever (RR=1.01; 95% CI=0.97 to 1.06; P=0.62), cough (RR=1.07; 95% CI=0.85 to 1.35; P=0.55), expectoration (RR=1.05; 95% CI=0.51 to 2.16; P=0.90), headache (RR=1.02; 95% CI=0.28 to 3.75; P=0.97), or diarrhoea (RR=1.90; 95% CI=0.74 to 4.88; P=0.18) between ICU group and non-ICU group.

### Complications for exacerbation

**Figure 3:**
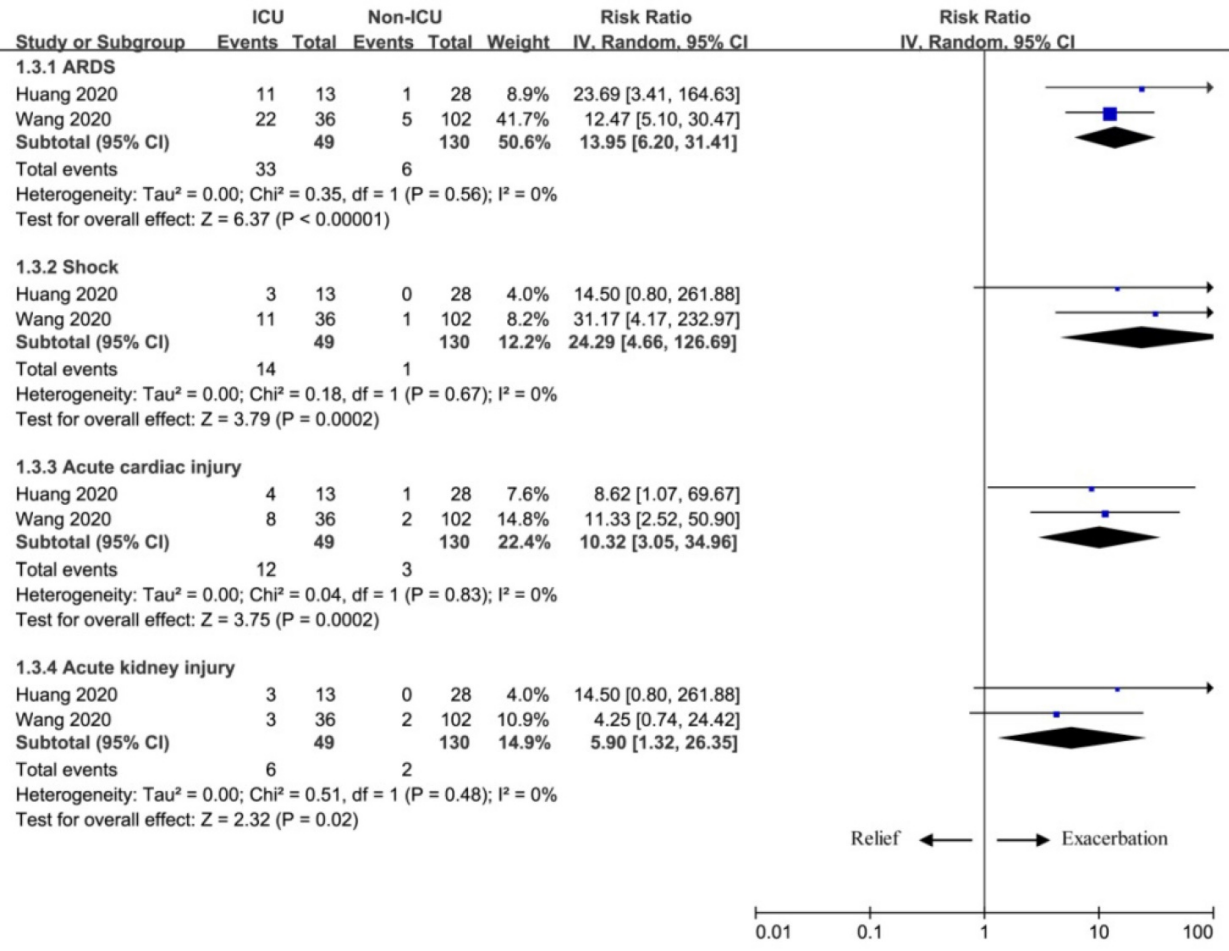
The influence of complications on exacerbation.

Shock, ARDS, acute cardiac injury and acute kidney injury commonly occurred as the exacerbation of NCIP. Our meta-analysis also revealed the potential in predicting the exacerbation of pneumonia based on the results of ARDS (RR=13.95; 95% CI=6.20 to 31.41; P<0.00001), shock (RR=24.29; 95% CI=4.66 to 126.69; P=0.0002), acute cardiac injury (RR=10.32; 95% CI=3.05 to 34.96; P=0.0002), and acute kidney injury (RR=5.90; 95% CI=1.32 to 26.35; P=0.02) between two groups (Figure 3).

## Discussion

A descriptive study reported 99 cases of NCIP from Wuhan Jinyintan Hospital from Jan 1 to Jan 20, 2020, and demonstrated that older men with comorbidities was more likely to suffer from NCIP and ARDS. The difference in clinical characteristics between severe and non-severe cases was not explored 17. In contrast, the proportion of men and women showed no statistical difference between ICU patients and non-ICU patients in another study 12. This inconsistency might be attributed to that the most of the affected patients in the previous report were male workers with exposure to Huanan Seafood Wholesale 12. Our results indicated that gender and Huanan Seafood Wholesale Market exposure showed no notable effect on the severity of these pneumonia patients.

That study also confirmed that the patients in ICU group were older and had more comorbid diseases than those patients in non-ICU group 12. Older age and comorbidity may be risk factors for the exacerbation of NCIP 17. The old age limit ≥65 years may be defined as the risk factor for exacerbation of COVID-19 (P<0.001) 20. Regarding the comorbidity in this meta-analysis, hypertension, cardiovascular diseases and COPD were revealed as the three highest risks for the exacerbation of NCIP, but diabetes, chronic liver disease and malignancy appeared to have no obvious effect on exacerbation of these patients.

Signs and symptoms may help identify the exacerbation and poor outcome of NCIP patients. Some symptoms such as dyspnea, abdominal pain and anorexia were found to be more common in ICU patients than those in non-ICU patients 10, 12. In our meta-analysis, dyspnea, myalgia or fatigue may be the high risk to predict the severity of NCIP patients, but fever, cough, expectoration, headache and diarrhoea demonstrated no substantial effect on the exacerbation of disease.

The most common laboratory abnormalities were reported to be depressed total lymphocytes, prolonged prothrombin time, and elevated lactate dehydrogenase, which might be associated with cellular immune deficiency, coagulation activation, myocardia injury, hepatic injury, and kidney injury 12. With the ongoing exacerbation of NCIP, ARDS, shock, acute cardiac injury and acute kidney injury might be the most common complications based on the results of this analysis.

Patients with low immune function such as old age, obesity, presence of comorbidity, HIV infection, longterm use of immune-suppressive agents and pregnant women may have improved mortality 21. Prompt administration of antibiotics to prevent infection and immune support treatment may reduce the complications and mortality of these patients 17. The reduced lymphocytes were found in most patients, suggesting that 2019nCoV mainly damaged lymphocytes, especially T lymphocytes, which was similar to SARSCoV. Substantially decreased T lymphocytes might be an important factor for predicting the exacerbations of patients 22. The transmissibility of COVID-19 was similar to that of SARS-CoV in the range of 2.9-3.3 23. The overall adjusted case fatality rate (CFR) is estimated to be 3.06% for the COVID-19, which was lower than those of SARS-CoV (9.2%) and MERS-CoV (34.4%) 13.

This meta-analysis has several potential limitations. Firstly, there are only two cohort studies included and total 179 patients are used for the analysis. More studies with large sample should be conducted to investigate this issue. Secondly, this analysis aims to explore the risk factors for exacerbation prediction of NCIP patients based on the epidemiological and clinical features between ICU patients and non-ICU patients, but there is no clear definition of ICU admission in two included studies, which may produce some bias. Thirdly, significant heterogeneity is observed for diabetes, which may be caused by small sample, different requirement and conditions of ICU admission.

In conclusion, this study reveals several risk factors (e.g. hypertension, cardiovascular diseases, COPD, dyspnea, myalgia or fatigue) for early identification of critical cases in patients with NCIP. ARDS, shock, acute cardiac injury and acute kidney injury may be the most common complications for severe cases. These findings are of crucial importance for timely treatment and the improvement of prognosis.

## Figures and Tables

**Table 1:** Characteristics of included studies

Study 1: Wang 2020						Study 2: Huang 2020				
Group	Number	Age, median (IQR)	Male	Female	Huanan seafood market exposure (n)	Number	Age, median (IQR)	Male (n)	Female (n)	Huanan seafood market exposure (n)
ICU	36	66 (57-78)	22	14	5	13	49.0 (41.0-61.0)	11	2	9
Non-ICU	102	51 (37-62)	53	51	7	28	49·0 (41.0-57.5)	19	9	18
P value	-	<.001	0.34		0.3	-	0.60	0.24		0.75

IQR, interquartile range; ICU, intensive care unit.
